# Cryogel Scaffolds for Tissue-Engineering: Advances and Challenges for Effective Bone and Cartilage Regeneration

**DOI:** 10.3390/gels9120979

**Published:** 2023-12-14

**Authors:** Vito Cosimo Carriero, Laura Di Muzio, Stefania Petralito, Maria Antonietta Casadei, Patrizia Paolicelli

**Affiliations:** Department of Drug Chemistry and Technologies, Sapienza University of Rome, 00185 Rome, Italy; vitocosimo.carriero@uniroma1.it (V.C.C.); laura.dimuzio@uniroma1.it (L.D.M.); stefania.petralito@uniroma1.it (S.P.); mariaantonietta.casadei@uniroma1.it (M.A.C.)

**Keywords:** cryogels, scaffolds, tissue-engineering, bone reconstruction, cartilage reconstruction

## Abstract

Critical-sized bone defects and articular cartilage injuries resulting from trauma, osteonecrosis, or age-related degeneration can be often non-healed by physiological repairing mechanisms, thus representing a relevant clinical issue due to a high epidemiological incidence rate. Novel tissue-engineering approaches have been proposed as an alternative to common clinical practices. This cutting-edge technology is based on the combination of three fundamental components, generally referred to as the tissue-engineering triad: autologous or allogenic cells, growth-stimulating factors, and a scaffold. Three-dimensional polymer networks are frequently used as scaffolds to allow cell proliferation and tissue regeneration. In particular, cryogels give promising results for this purpose, thanks to their peculiar properties. Cryogels are indeed characterized by an interconnected porous structure and a typical sponge-like behavior, which facilitate cellular infiltration and ingrowth. Their composition and the fabrication procedure can be appropriately tuned to obtain scaffolds that match the requirements of a specific tissue or organ to be regenerated. These features make cryogels interesting and promising scaffolds for the regeneration of different tissues, including those characterized by very complex mechanical and physical properties, such as bones and joints. In this review, state-of-the-art fabrication and employment of cryogels for supporting effective osteogenic or chondrogenic differentiation to allow for the regeneration of functional tissues is reported. Current progress and challenges for the implementation of this technology in clinical practice are also highlighted.

## 1. Introduction

Currently, non-healing bone fractures or articular cartilage injuries resulting from accidents, surgical resection, age-related pathologies, infections, or cancer have an elevated epidemiological and clinical incidence [1,2]. These kind of injuries consist of non-union fractures in the case of skeletal bones, or age-related or accidentally damaged articular joints. In both cases, they can be considered as wounds which are unrepairable through physiological mechanisms. Since damage of these tissues is associated with a loss of structural function, inflammation, and extreme pain, it is fundamental to replace or repair such injuries and limit the consequent debilitation [2]. In clinical practice, graft implantation is a technique commonly adopted to fill gaps or substitute defective tissues to recover the native shape and physiological function of a specific compartment [3]. In particular, autologous, allogenic, xenogenic, or synthetic grafts are used for the scope. However, the healing potential of these traditional strategies is often limited by relatively low accessibility, which may occur for autologous grafting, as well as high costs, infection potential, risk of rejection, and a low success rate for allogenic, xenogenic, and synthetic grafts [1].

In recent years, an innovative field of regenerative medicine proposed a valid and promising alternative to traditional autologous or allogenic grafting. The aim of tissue-engineering is indeed to reconstitute damaged human tissues through the use of biocompatible materials, in the form of scaffolds, autologous cells, and growth-stimulating factors. These three elements are generally referred as the tissue-engineering triad.

Among recently reported scaffolds, polymer-based networks represent a suitable and interesting approach for tissue-engineering, including the regeneration of complex and highly specialized bone and cartilage tissues [4]. In particular, polymer scaffolds characterized by highly porous networks, such as cryogels and aerogels, have resulted particularly promising for tissue regeneration, as they can facilitate cell distribution within the scaffold. Cryogels represent a technical evolution of hydrogels, a three-dimensional network of hydrophilic polymers which share the same chemical properties, but differ for the internal structure. Both hydrogels and cryogels possess a porous inner structure; however, hydrogels are characterized by small, isolated, and not interconnected pores [5]. Such features of hydrogels do not allow proper cellular seeding, spreading, and proliferation [1]. On the contrary, cryogels consist of a macro-porous and interconnected network, comparable to a sponge. The internal structure of cryogels offers larger available free space for cell hosting, and generally confers the suitable mechanical properties for tissue reconstruction [6]. Like cryogels, aerogels are highly porous systems, characterized by a very low density and thermal insulation properties [7]. Aerogels are usually produced via a supercritical drying process, in which the liquid phase is removed by a fluid in supercritical conditions [8]. Both cryogels and aerogels have been proposed as scaffolds in tissue-engineering thanks to their peculiar porosity and high specific surface area. Indeed, a porous and largely interconnected structure represents a key property of a scaffold in order to allow effective tissue regeneration. On the one hand, a macro-porous network facilitates homogeneous cell infiltration, thus enabling colonization of the entire scaffold [1]; on the other hand, it allows unhindered diffusion of nutrients and metabolic waste during the cell life cycle [9,10]. Although aerogels and cryogels share similar potential for tissue-engineering application, the use of aerogels in this area is beyond the scope of this revision; therefore, our discussion focuses only on the advances and challenges of cryogel-based tissue-engineering.

Several in vitro and in vivo studies confirmed the high potential and applicability of cryogels in the field of orthopaedic non-healing injuries [11]. An interesting feature of this technology applied to bone and joint regeneration is the possibility of tailoring the physical, mechanical, chemical, and biological properties of scaffolds to closely match those of osteon- and chondro-like tissues to allow for their proper reconstruction. The use of cryogels has the advantage of finely tuning all these properties with proper selection of specific scaffold components as well as with accurate control of critical fabrication parameters. Lastly, they can be fabricated according to a specific shape and dimension, which can precisely fit with a defect site [1,4,12].

**Figure 1:**
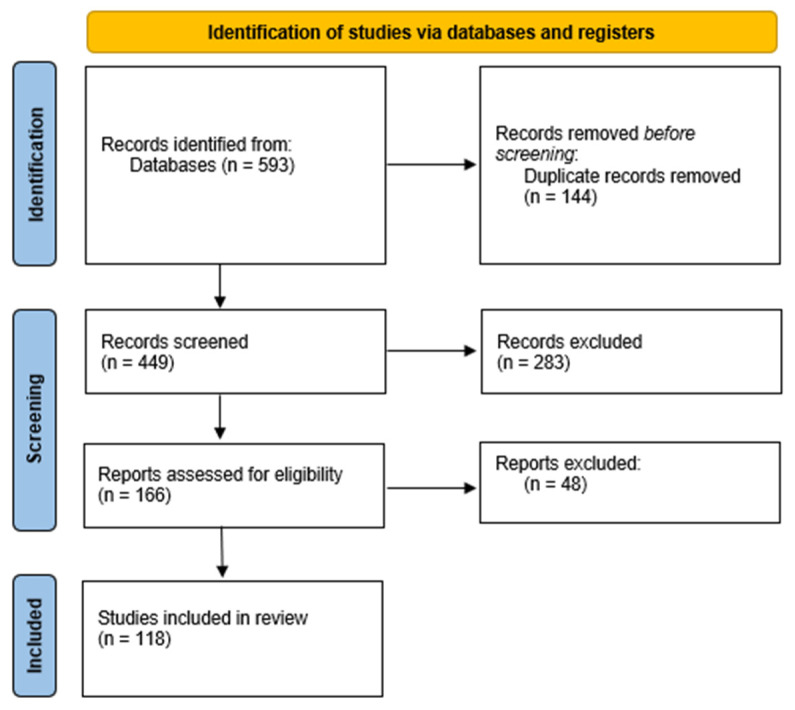
PRISMA 2020 flow diagram for systematic reviews. Keywords used for research: cryogels; tissue-engineering or reconstruction; bone; osteoblasts; osseous; osteogenesis; cartilage; chondrocytes.

Therefore, cryogel scaffolding represents a promising strategy for repairing damaged bones or articular joints. The most recent results achieved in this field will be here reviewed and discussed according to the PRISMA statement for systematic reviews [13], as reported in Figure 1.

## 2. Bone and Cartilage Reconstruction with Cryogel Scaffolds

**Figure 2:**
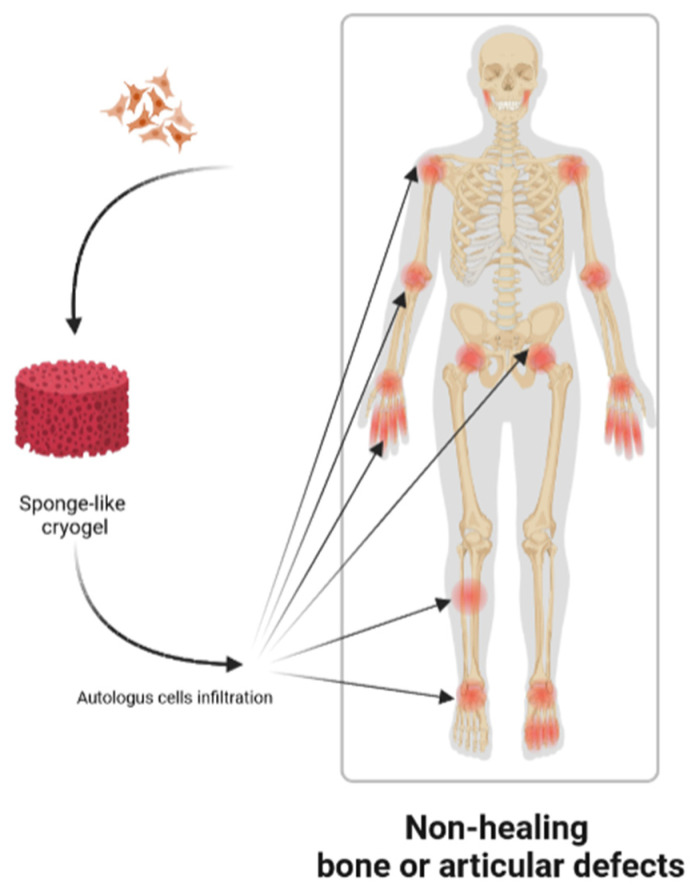
Use of cryogels as scaffold for non-healing and critical-sized joint or bone defects.

The aim of cryogel-based orthopedic tissue-engineering is to regenerate bone or cartilage tissues, starting from a three-dimensional culture of undifferentiated cells supported by a sponge-like scaffold. To reach this goal, autologous cells are collected directly from the patient and subsequently seeded on a cryogel, as schematized in Figure 2.

The role of a scaffold, along with the other elements of the tissue-engineering triad, is to support cell adhesion, migration, growth, differentiation, and guide the development of a complex histological system [14]. Tissue regeneration can be performed both in vivo and ex vivo; in any case, a scaffold is implanted into the patient at the site of the non-healing gap. Successful histological integration is obtained by progressive substitution of the supporting scaffold by a functional and histo-integrated tissue [15]. For this reason, it is necessary to fabricate a scaffold that is perfectly compatible and integrable with the host environment, non-immunogenic, and potentially able to regenerate a specific tissue while totally restoring its function [15,16]. In addition to biocompatibility and biodegradability, a scaffold must be tailored according to specific tissue characteristics, which represents a particularly demanding challenge in the case of load-bearing tissues, such as cartilage and bone [17,18]. In order to allow the regeneration of a functional tissue at the wound site, a scaffold should possess proper mechanical competence and stiffness to substitute the damaged tissue and positively influence cell differentiation [14].

These considerations point out that the materials used for cryogel fabrication as well as the fabrication process itself need to be properly evaluated and tuned to allow for the effective clinical translation of cryogels.

## 3. Cryogel Fabrication

**Figure 3:**
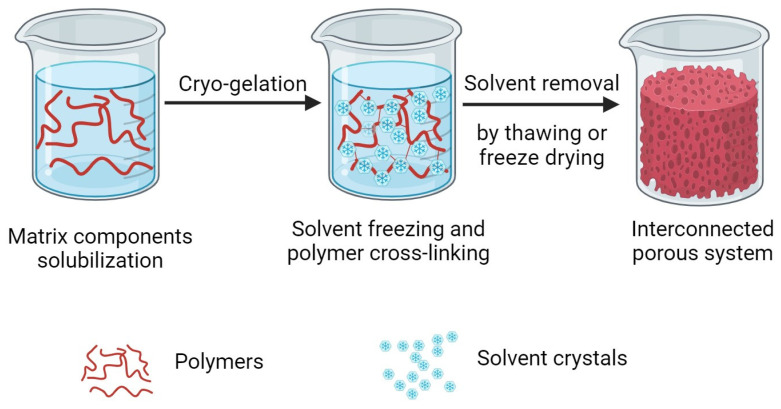
Schematic of the procedure used for cryogel fabrication.

The characteristic inner structure of cryogels is obtained following a pore-templating procedure that uses solvent crystals as porogens [17]. All the procedures reported in the literature share a common sequence that is composed of three different steps, as shown in Figure 3 [18,19,20]:

1. Solubilization of the matrix components, which can be represented by monomers or macromers, and the cross-linking agents;
2. Cryo-gelation;
3. Solvent crystals removal.

As suggested by the name cryogel, polymer gelation is performed at sub-zero temperatures. In fact, after the solubilization of monomers or macromers, the solution is chilled under the freezing point of the solvent, which is usually represented by water. Under these conditions, water solidifies and the formed ice crystals act as a template for the inner open-pore structure of the resulting cryogel [15]. After the beginning of crystal nucleation, polymerization of the monomers or macromers and cross-linking occurs, building the pore walls around the ice crystals. Once the polymer cross-linking is completed, the solvent crystals are removed, leaving behind an interconnected continuous pore network as a negative replica [1]. In the following paragraphs, the critical parameters influencing each step of the procedure used for the fabrication of cryogels intended for bone or cartilage reconstruction will be discussed.

### 3.1. Solubilization of the Matrix Components

In the first step, an aqueous solution of the matrix components is prepared. Like hydrogels, cryogels are usually obtained by the physical or chemical cross-linking of polymers. Both natural and synthetic polymers can be used for chondrogenic and bone tissue regeneration. The polymers most frequently investigated in this research area will be explored in Section 4.

### 3.2. Freezing and Cryo-Gelation

Freezing and cryo-gelation steps are crucial for the architecture and topography of cryogels and, therefore, for their mechanical properties. During the cryo-gelation step, the morphology and size of the scaffold pores critically depend on the proper balance between the freezing rate and the gelation kinetics [1,9,15].

**Figure 4:**
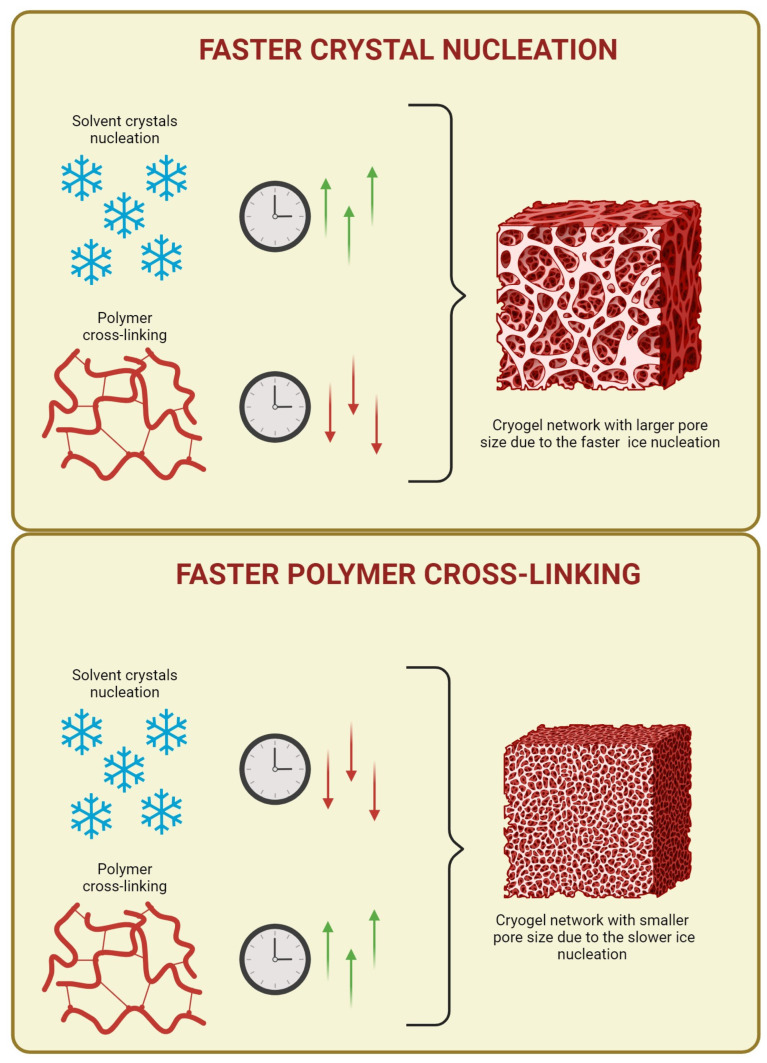
Effect of the freezing and gelation kinetics on the pore size of the resulting porous network. ↑ and ↓ mean faster and slower kinetics, respectively.

The process consists of a combination of two events, which allows for the formation of a porous network. Specifically, cooling of the precursor solution at a sub-zero temperature induces solvent solidification, and, in this way, two micro-phases are constituted: frozen regions and un-frozen regions [15]. The frozen regions are composed of solvent crystals, which act as porogens, while the un-frozen microphase is where cryo-gelation takes place, or rather the polymer chains cross-link around the ice crystals and build the pore walls [15,21]. These two events (solvent crystallization and polymer cross-linking) must occur in a precise hierarchy, as schematized in Figure 4. In particular, solvent nucleation and crystallization must take place before polymer cross-linking [15]. When this occurs, all the monomers, macromers, and cross-linking reagents of the starting solution undergo a cryo-concentration or freeze-concentration effect [21,22], which is one of the major factors responsible for the increase in the rate of the chemical reactions and intensification of intermolecular interactions inside the un-frozen microphases. In other words, these un-frozen microphases act as microreactors [1,9,22]. Indeed, during the freezing process, solvent solidification subtracts water molecules from the un-frozen phase, thus increasing the concentration of the solutes. Therefore, before freezing is completed, some chemical reactions can occur and proceed at rather fast rates despite the low temperature. Alongside this aspect, it is also necessary to consider the cooling rate at which the cryo-gelation process is carried out, as it has a direct impact on the ice crystal habits formed, such as size, number and morphology, and this, in turn, impacts the pore size of the polymer network, once the ice crystals are removed. In particular, very high cooling rates lead to the formation of many small ice crystals and a corresponding polymer network with a hydrogel-like structure. In contrast, a small number of ice nucleation sites, which yields few large ice crystals, is obtained when the freezing process is carried out at slower rates of freezing [15]. The second event allows for the formation of polymer networks characterized by dense and resistant pore walls and properly named cryogels. This result can be usually achieved when the freezing stage is carried out within a range of −5 °C to −20 °C [9,23,24,25,26,27,28,29].

It is also known that ice crystal morphology is a function of the cooling rate. Specifically, samples frozen at slow freezing rates yield dendritic ice crystals, whereas samples frozen at fast freezing rates produces needle-like structures [30]. Also, this factor, evidently, contributes to the determination of the properties and behavior of the resulting polymer network. Therefore, all that considered, the cryo-gelation step requires a subtle balance between the operating conditions and the composition of the precursor solution, as they have a tremendous effect on the structure and properties of the resulting cryogel [15].

In conclusion, cryotropic gelation is a delicate interplay between solvent crystallization and polymer cross-linking. The precise sequence and rate of these events significantly influence the final cryogel structure. Therefore, understanding and careful handling of these parameters is necessary to obtain cryogels with specific properties, which can emulate the complex structure of a physiological tissue.

### 3.3. Freezing Methods

The cooling process may be carried out using different techniques. Generally, the immersion of a mold in a freezing bath (FB) [31] or unidirectional freezing (UF) system [32] are used to freeze the precursor solution. In the first case, a temperature-controlled bath is used to freeze the sample at the desired temperature. Since the cooling process takes place with a centripetal direction, crystallization occurs entropically in the sample [33]. In contrast, by UF, it is possible to induce ice crystal formation in a preferential direction. Therefore, the resulting cryogel will show an internal structure characterized by oriented pores. The freezing direction can be controlled by immersing cooled pins of an inert material, such as copper, inside the precursor solution. When these pins are cooled down, solvent crystallization will occur radially along the pin axis. Due to the proximity of two consecutive pins, crystals elongation is limited. It means that in each section, so-formed cryogels are structured in macroscopic conic regions made by a lamellar pore network. Additionally, the upper and lower edges of the cryogels made by UF present a pore network tangential to the cryogel surface. This arcade composition of the inner structure is so composed of two border regions made by longitudinal fibres and one inner region composed of a lamellar network, oriented perpendicularly to the edges. The UF approach for freezing is a relatively new technique, which has been proposed to meet the peculiar and complex requirements of some specific tissues, such as the physiological structure of cartilage, which is structured in aligned lamellar fibrils. This freezing technique allows for the tuning of the mechanical behavior of a scaffold, such as to match that of native cartilage. Such a feature is particularly important for the regeneration of complex tissues, such as bone and cartilage, because the mechanical behavior of a scaffold influences morphology and expression pattern of seeded cells [32]. The correlation among scaffold properties, cellularity, and tissue regeneration will be further discussed in Section 5.

### 3.4. Polymer Cross-Linking

Cryogel formation may involve physical or chemical cross-linking among the polymer chains. However, physically cross-linked cryogels usually present some drawbacks associated with poor mechanical and physical properties, and, for this reason, they are barely proposed as scaffolds for tissue-engineering. Instead, chemical cryogels based on covalent bonds among polymer chains usually guarantees a more stable 3D network and better resistance to physical or mechanical stresses. Therefore, chemical cryogels appear to be superior scaffolds for tissue regeneration, as reported for cryogels made of chitosan and gelatin [15]. Indeed, physical cryogels composed of these two polymers resulted brittle and inhomogeneous. On the contrary, when the same polymers were covalently cross-linked, the corresponding cryogel networks presented higher mechanical stability and, in general, resulted more suitable to serve as a scaffold for cell seeding and growing. Such stability is also important because it allows for scaffold sterilization, even by moist heat. Sterilizability is a fundamental requirement for any biomedical use of cryogels [34], and steam sterilization represents the easiest, most effective, least expensive, largely employed, and FDA-approved method for the sterilization of this kind of structure [15,35].

**Figure 5:**
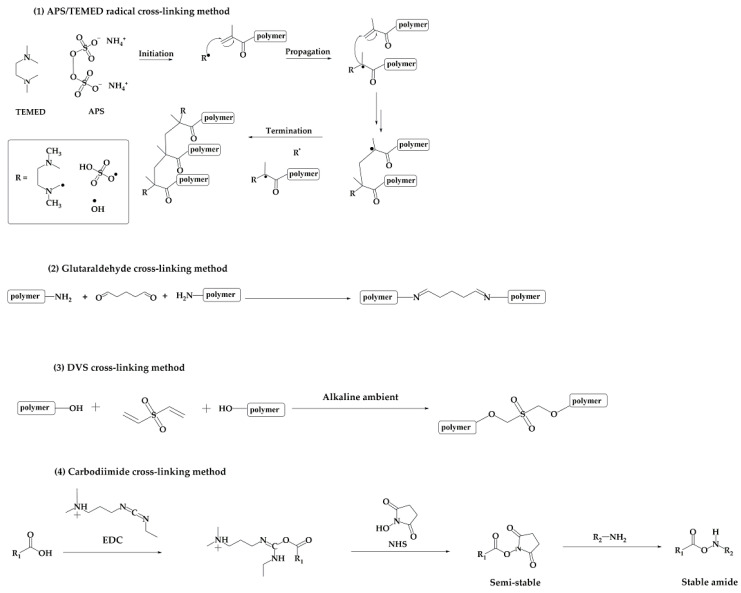
Schemes of the cross-linking reactions most frequently employed for cryogel fabrication. (1) APS/TEMED-induced radical cascade [36]; (2) glutaraldehyde-based cross-linking; (3) DVS-based cross-linking; (4) carbodiimide coupling reaction.

One of the most commonly used cross-linking approaches is based on the radical reaction of polymers carrying methacryloyl groups on their chains. The radical cascade is usually triggered with peroxides, such as ammonium peroxydisulfate (APS), a radical initiator, which is usually employed along with N,N,N’,N’-tetrametylethylendiamine (TEMED) [20,36,37,38], as reported in scheme 1 of Figure 5 [36]. The radical cross-linking of methacryloyl or, more generally, vinyl derivatives can also be induced via UV-photocuring. This cross-linking strategy is largely employed for hydrogel fabrication [39,40] and usually involves the use of photoinitiators, such as lithium phenyl-2,4,6-trimethylbenzoylphosphinate (LAP) [41] or 2-hydroxy-4′-(2-hydroxyethoxy)-2-methylpropiophenone (I2959), to initiate a radical cascade [42]. Some attempts have been made to adopt UV-induced polymer cross-linking for the fabrication of cryogels. To do this, the cryo-gelation step is usually divided into two consecutive phases. Firstly, the precursor solution undergoes a partial freezing, which allows for the germination of the solvent crystals, but not the full solidification of the sample. The partially frozen samples are then exposed to UV radiation to initiate the cross-linking process, before the ice crystallization stage is completed [43].

Other cross-linking protocols, largely used for the fabrication of cryogel scaffolds suitable for bone or cartilage regeneration, are based on coupling reactions with bifunctional agents, such as glutaraldehyde (GA) and divinyl sulfone (DVS) [15,44,45], reported, respectively, in scheme 2 and 3 of Figure 5. A GA coupling reaction can been used for the cross-linking of polymers bearing nucleophiles, such as primary amino groups of gelatin [15], collagen [46], and chitosan [19]. As an alternative, the same polymers can be chemically cross-linked using DVS in alkaline ambient. This cross-linker has been used to form stable polysaccharidic networks [46], as the hydroxyl groups of polysaccharides can react with the two electronic-deficient double bonds of DVS, leading to polymer cross-linking.

Another frequently used coupling reaction is known as carbodiimide coupling, reported in scheme 4 of Figure 5, based on the use of (1-ethyl-3-(3-dimethylaminopropyl)carbodiimide (EDC) along with N-hydroxysuccinimide (NHS) [33,47,48,49,50]. These reagents are used to couple carboxylic groups of one polymer with primary amino groups or hydroxyl groups of the same polymer or a different one. It works through the initial activation of the carboxylic groups, followed by their subsequent reaction with primary amino or hydroxyl groups. The EDC/NHS-mediated coupling reaction has been proposed for the cross-linking of gelatin and collagen [51].

The above-reported chemical cross-linking methods are largely employed for cryogel fabrication, even if some of the employed reactants and their residues raise important safety issues, as reported in Table 1, where the main advantages and disadvantages of these cross-linking methods have been summarized. For this reason, there is a continuous search for alternative and safe cross-linking reactions. Recently, the use of graphene oxide (GO) as cross-linker of gelatin-based scaffolds has been proposed. The cross-linking reaction involves the nucleophiles on the gelatin backbone, such as primary amino groups, and the different chemical functions on the surface and edges of GO sheets [24].

### 3.5. Solvent Crystals Removal

After the cryo-gelation step is completed, the solvent crystals can be removed by thawing at room temperature [5,15,52,53], or by freeze-drying [54,55,56]. In both cases, a porous structure, which constitutes a negative replica of the solvent crystals, is obtained.

## 4. Material Used for the Fabrication of Cryogels Suitable for Bone and Cartilage Reconstruction

As described in Section 2, scaffolds used for tissue regeneration should be made by polymers which possess precise histological and biological properties to allow proper cellular adhesion and tissue reconstruction. Moreover, the ideal cryogel components should be biodegradable, since according to the purpose of regenerative medicine, gradual substitution of a cryogel network by natural histological material is expected [12]. This means that a non-healing injury will be finally substituted by biological tissue, which will possess the required mechanical properties to support the load-bearing function of bone tissue and the mechanical articulation of joint cartilage.

Finally, a scaffold matrix should reproduce the mechanical and physical qualities of the target tissue, i.e., adequate hardness for bone and specific tribological behavior in the case of articular cartilage [36,57,58]. Nevertheless, reproduction of the complex structural and functional properties of biological tissues is very difficult with the employment of a single material [2,33]. For this reason, to obtain suitable scaffolds, cryogels are frequently fabricated with the blending together of different materials, including both organic and inorganic biomaterials, as summarized in Table 2 and Table 3. When two different biopolymers are combined for the production of a cryogel scaffold, they can be cross-linked with each other to form a continuous and interconnected network [9], or they can be separately cross-linked to form an interpenetrated network [59]. The construction of interpenetrated networks is a fine strategy which allows us to obtain more complex and mechanically resistant systems. Indeed, besides to the possibility of blending different polymers with different chemical or biological properties, these particular networks possess the advantage of tailoring and customizing the porosity and the mechanical behavior of a final system [60]. The fabrication of these systems involves the recurrent synthesis of a new cryogel structure inside the macro-porous meshes of a pre-formed cryogel network [61]. This protocol can be applied to introduce a second or more polymeric networks within the first cryogel [59].

Cryogel scaffolds for bone and cartilage regeneration have been prepared from both natural and synthetic polymers. The latest advances made with biomaterials used for cryogel fabrication will be discussed in the following paragraphs.

### 4.1. Collagen-Based Cryogels

Collagen (Coll) is a natural component of the extracellular matrix (ECM) and is largely represented in all mammalian tissues. It contains amino acid sequences, which play a tremendous role in cell adhesion and ECM degradation and remodeling [33]. These sequences include the tripeptide arginine–glycine–aspartate (RGD), which has been shown to mediate cell adhesion by interaction with cell integrins, which is a fundamental step for the first-phase of cell colonization of a scaffold [62]. For these reasons, Coll has been largely used for bone and cartilage regeneration, alone or in combination with other organic or inorganic materials. In this sense, Odabas et al. developed a cryogel system combining Coll with carboxymethyl cellulose (CMC) and tricalcium phosphate (TCP). CMC has been proposed as it can mimic aggrecan, an ECM protein which provides gel behavior to cartilaginous or membranous bone tissue [33]. TCP, instead, is usually used to improve the mechanical properties of a scaffold, while providing it with an osteoinductive property, which is the ability to induce differentiation into osseous tissue. Particularly, the addition of TCP to Coll/CMC cryogels caused an increase in compressive resistance from 300 kPa for plain Coll/CMC cryogels to more than 400 kPa for TCP-enriched systems [33]. Although TCP is able to improve the mechanical properties of cryogels, we are still far from those of native bone and cartilage tissues, which are characterized by compressive moduli in the range of 10–20 GPa for bone [63] and 0.5–1.1 MPa for cartilage [64]. Therefore, other strategies have been attempted to obtain cryogel scaffolds that are more suitable from a mechanical point of view. This issue is of fundamental importance for tissue-engineering in general, but it is particularly challenging for the reconstruction of load-bearing tissues, such as bone and cartilage. Indeed, there is continuous research aimed at improving the mechanical competence of scaffolds used for the regeneration of these tissues [26]. This feature is also important because it has been observed that the stiffness of a scaffold influences its osteoinductive nature, and thus its ability to induce mesenchymal cells to differentiate into mature bone-forming cells [65]. In this context, several works explored the use of hydroxyapatite (HA) (Ca_10_(PO_4_)_6_(OH)_2_), which is a major component of the mineral part of bone, and it has been largely employed as an inorganic additive for cryogels [61,62,63,64,65,66,67,68,69,70].

### 4.2. Gelatin-Based Cryogels

Coll, as a natural component of the ECM, would represent an ideal candidate for cryogel fabrication; however, its actual use is hindered by its low solubility in water. Indeed, harsh acidic conditions are usually needed to allow the preparation of Coll-based cryogel scaffolds [71,72]. For this reason, gelatin (Gel), a derivative produced from the partial hydrolysis of Coll, is most frequently used for the development of scaffolds for tissue regeneration. Compared to native Coll, Gel shows higher solubility in water, while preserving the typical RGD sequences of Coll, and so possesses the innate properties that promote cellular adhesion [38]. Nevertheless, Gel-based scaffolds possess two major drawbacks: weak mechanical properties and poor thermal stability. To overcome these limits, Gel is frequently chemically cross-linked and combined with other polymers. In particular, to improve the thermal stability, Gel has been chemically cross-linked with GA [73,74,75,76]. However, due to the toxicity of this cross-linking agent, alternative dialdehydes have been proposed to produce stable and mechanical-resistant Gel-based networks, such as pullulan dialdehyde [26] and dextran dialdehyde [6], obtained from the oxidation of pullulan and dextran, respectively. Both these polymers can react with Gel via a Schiff’s base reaction, thus forming stable networks.

Other strategies used to produce Gel-based chemical cryogels involve the modification of the gelatin backbone with specific chemical moieties. One of the most diffuse functionalizations of Gel is based on the insertion of methacryloyl groups, with the consequent formation of methacryloyl Gel (GelMA) [77]. This derivative allows for the production of stable, three-dimensional networks, with mechanical properties and porosity tuneable by varying the derivatization degree of GelMA [37,38,78], which, however, is sometimes not appropriately determined [79] or even non evaluated at all. Despite this feature, GelMA is rarely used alone for the fabrication of cryogel scaffolds, but it is usually mixed with other biomaterials in order to achieve scaffolds with adequate mechanical resistance for tissue regeneration [80]. Nevertheless, Di Muzio et al. recently have reported on the possibility of obtaining cryogels made of GelMA only, characterized by high biocompatibility and undisputable mechanical resistance [9]. These results could be achieved using GelMA derivatives characterized by a very low physical gelation point. They supposed, indeed, that if GelMA physical gelation occurs too early during the cryo-gelation procedure, it may hinder the covalent cross-linking of the polymeric chains and lead to networks with poor mechanical competence, which usually require the addition of mechanical adjuvants. GelMA derivatives with low gelation temperatures can be obtained using Gel obtained from alternative animal sources, such as cold-water fish, as starting material [81] or by adopting a specific synthetic procedure carried out under denaturing solvent conditions [79].

When Gel-based scaffolds are used for bone tissue regeneration, they are often decorated by osteoinductive components [38], such as silica bioglass, which furnishes the inorganic ions necessary to support bone mineralization [82] and provides an ECM-like environment that enhances osteo regeneration during the bone healing process [38].

### 4.3. Silk Fibroin-Based Cryogels

Silk fibroin (SF) is a fibrous protein obtained from both silkworms like *Bombyx mori* and orb-weaving spiders like *Nephila clavipes* [83]. It is able to form peculiar gels when solubilized in water. SF chains are characterized by hydrophilic domains and hydrophobic β-sheet-crystallites [84]. While hydrophobic domains provide the gel with mechanical strength and resistance, hydrophilic regions guarantee its wettability and regulate its elasticity and toughness. [29,60]. The resulting combination of stiffness and resistance make SF an interesting material for the fabrication of scaffolds for tissue regeneration, and, more specifically, bone tissue-engineering. In this context, stable and mechanically resistant SF-based gels have been developed using ethylene glycol diglycidyl ether as a cross-linking agent. This cross-linker is able to trigger the conformational transition of fibroin from random coil to ordered β-sheet structure, which is responsible of fibroin gelation. SF-based cryogels obtained in this way show a unique elasticity feature that allows them to resist complete compression without any crack development. Compressed cryogels immediately swell during unloading to recover their original shape [84]. Instead, Yetiskin and colleagues studied the design of a silk-fibroin interpenetrated system, which allowed the formation of scaffolds with improved mechanical competence [60].

### 4.4. Glycosaminoglycans (GAGs)

GAGs are a class of natural polymers frequently employed for the fabrication of scaffolds for bone and cartilage regeneration, as they are components of the ECM proteoglycans. GAGs are linear polysaccharides made up of repeating disaccharide units. Among them, chondroitin sulfate (CS) and hyaluronic acid (Hac) are the GAGs most often explored in the field of tissue regeneration.

Chondroitin sulfate (CS) is a sulfated polysaccharide based on a chain of alternating units of glucuronic acid and N-acetylgalactosamine. It is an essential component of cartilage and plays an important role in the elasticity and functionality of articular cartilage; moreover, it offers stimulus for proteoglycan and type II collagen secretion [20]. Such features make CS an interesting biomaterial for the fabrication of scaffolds aimed to cartilage regeneration. This GAG exists in various forms related to the nature of the disaccharide unit, sulfation degrees and molecular weight [85]. Sulfation can occur on either O-4 or O-6 position, giving CS-A and CS-B respectively. Commercially, it is available as variously composed mixtures of CS-A, CS-B and a minor fraction of un-sulfated chondroitin [86,87]. Nevertheless, some works have been published reporting the use of pure CS-4-sulfate [88] or CS-6-sulfate [89].

HAc is a non-sulfated GAG composed of disaccharide repeating units consisting of D-glucuronic acid and N-acetyl-D-glucosamine. It is mostly found in the ECM of connective tissues, where it supports cell migration and proliferation as well as cartilage integration throughout the development of embryonic cartilage [45,90,91].

**Figure 6:**
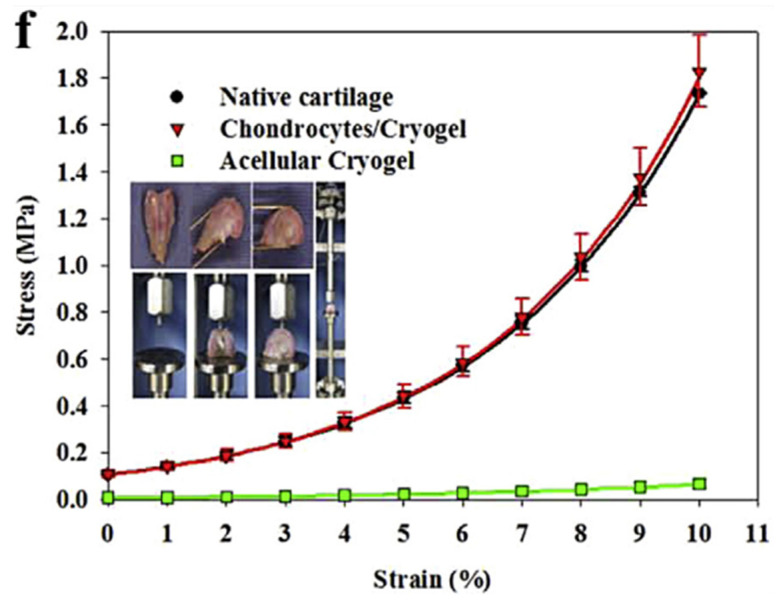
Comparison of the stress–strain curves of native cartilage, acellular cryogel, and chondrocytes-seeded cryogel, 3 months post-implantation. Cryogels were made of chitosan/gelatin/chondroitin-6-sulfate/hyaluronan. Reproduced with permission from [89].

GAGs have rarely been employed alone for cryogel fabrication, whereas they are frequently combined each other or with other biomaterials to closely mimic natural ECM properties and behavior. In this sense, Kuo and colleagues prepared macro-porous gelatin/chondoitin-6-sulfate/hyaluronan cryogel scaffolds for cartilage tissue-engineering [89]. Such cryogels proved to be effective for emulating the extra-cellular physiological environment and exerting stimuli for cartilage reconstruction. However, the inclusion of chitosan as a further cryogel component resulted in a general improvement in the biomechanical properties of the scaffold. Chitosan is a cationic (1-4)-2-amino-2-deoxy-β-D-glucan polysaccharide, which is generally considered to resemble the structure of GAG and is believed to mimic its function in the modulation of cell behavior and phenotype, as observed by Kuo and colleagues. These authors evidenced the necessity of blending multiple different biopolymers to mimic as much as possible the complex physiological environment of cartilage, while promoting cell proliferation and differentiation, in order to allow the formation of mechanically functional cartilage, as pointed out by the stress–strain curves reported in Figure 6 [89].

### 4.5. Other Natural Polymers

Among natural polymers, other polysaccharides have been proposed for the fabrication of cryogel scaffolds for bone and cartilage regeneration. For example, chitosan appears to be a good candidate for promoting chondrogenic differentiation due to its structural similarity to GAGs [89]. As largely mentioned in the above sections, the reproduction of the native ECM positively influences cell growing and differentiation. For this reason, chitosan has been blended with other natural polymers in order to produce cell-friendly scaffolds. More specifically, chitosan has been included in Gel-based, CS-based, or HAc-based cryogels [15,44,49], and it has also been proposed in combination with silk fibroin [18] or agarose [92,93].

Other natural polymers employed for the fabrication of cryogels intended for bone or cartilage reconstruction include alginate (Alg), dextran (Dex), and nanocellulose (nCell). Alg is a natural polyuronate (polymer composed of uronic acids) derived from brown seaweeds and some bacteria. Alg has been proposed to produce scaffolds for tissue-engineering purposes because it is capable of forming stable gels when exposed to multivalent cations through the formation of characteristic egg-box structures [94]. Dex is a bacteria-derived polysaccharide, which shows enzymatic degradation and good biocompatibility [12]. Bölgen and colleagues developed dextran-based cryogels, using Dex as an HEMA-L-lactate derivative, obtaining an interconnected network, which demonstrated compatibility with cartilaginous cells. Regarding mechanical behavior, the tested systems showed remarkable mechanical flexibility, softness, and toughness that might simulate tissue softness [95].

nCell, instead, has been proposed as a candidate for tissue-engineering due, in particular, to the mechanical properties of its scaffolds [96]. It exhibits good micro-structuring through the formation of weak interactions (H-bonds, Van der Waals interactions, etc.) among the polymeric chains [97]. nCell-based cryogels meet the microstructure and mechanical performance requirements of scaffolds for tissue-engineering. However, when proposed for bone tissue regeneration, a limiting factor in nCell utilization is the lack of bioactivity to induce proper bone regeneration. Indeed, nCell alone shows poor osteoconductivity; therefore, it must be integrated with bioactive compounds, such as silica bioglass, to promote bone tissue formation and mineralization [98].

Most of the polymers reported in this paragraph show good biocompatibility properties and other interesting features for cryogel fabrication, but they generally lack structural motifs, which may allow cell adhesion and thus promote scaffold cellularity. For this reason, they can be barely used alone for the fabrication of cryogels intended for tissue-engineering purposes, whereas they are frequently proposed in combination with other biopolymers [55].

**Table 2:** Natural materials employed for bone or cartilaginous tissue reconstruction via cryogel scaffolding.

Principal Cryogel Component	Key Properties	Blended Organic Materials	Inorganic Additives	Cross-Linking Method	Refs.
Collagen	ECM component; RGD sequences	gelatin; elastin		EDC/NHS	[51]
		carboxymethyl cellulose	TCP	physical cross-linking	[33]
			HA	EDC/NHS	[71]
		keratin	silica	EDC/NHS	[99]
			HA	GA	[46]
		hyaluronic acid methacrylate		GA	[32]
		chitosan; fucoidan; chondroitin sulfate		physical cross-linking	[2,100]
Gelatin	ECM emulator; RGD sequences; MMPs binding sites; stiffness	chitosan		GA	[15]
		hyaluronic acid		EDC/NHS	[50]
		DexOx	HA	Schiff base reaction	[101]
		p-HEMA		APS/TEMED	[102]
		DexOx	HA	Schiff base reaction	[6]
		hyaluronic acid; chondroitin sulfate; chitosan		EDC/NHS	[89]
			HA	EDC/NHS	[70]
				GA	[74]
				GA	[75]
			HA	GA	[76]
			bioglass	APS/TEMED radical cross-linking	[38]
		bone morphogenic protein-2		APS/TEMED radical cross-linking	[77]
		poly(lactic-co-glycolic acid)	HA	EDC/NHS	[66]
			HA	EDC/NHS	[103]
		heparin		EDC/NHS	[48]
				UV photocuring	[39]
		BMP-2 biomimetic peptide and VEGF		APS/TEMED	[37]
		hyaluronic acid; chondroitin sulfate		EDC/NHS	[88]
		carboxymethyl chitosan		EDC/NHS	[49]
				APS/TEMED radical cross-linking	[9]
		alginate		Ca(II) DVS	[55]
Silk fibroin	strength; toughness; mechanical stress resistance; mechanical flexibility;			1,4-butanediol diglycidyl ether (BDDE) or ethylene glycol diglycidyl ether (EGDE) and TEMED;	[29]
				BDDE/TEMED	[60]
				N,N′-methylene-bis(acrylamide) (MBAm) or N,N,N′,N ′tetramethylenediamine (TMDA); APS/TEMED radical cross-linking	[12,95,104,105,106]
Chondroitin sulfate	mechanical flexibility; stiffness; toughness; biological trigger for cellular differentiation	hyaluronic acid		APS/TEMED cross-linking on methacryloyl derivative	[20]
		gelatin; chitosan		APS/TEMED radical cross-linking	[19]
Hyaluronic acid	mechanical flexibility; stiffness; toughness; biological trigger for cellular differentiation; hydrophilicity	gelatin		EDC/NHS	[107]
		methoxy-PEG-acrylate or RGD sequences		APS/TEMED radical cross-linking	[90]
Chitosan	mechanical flexibility	Agarose; gelatin		GA	[92]
			biphasic calcium phosphate	EDC/NHS	[25]
		gelatin; chondroitin sulfate; hyaluronic acid		EDC	[89]
		gluconic acid		EDC/NHS	[17]
		Silk fibroin	Ag/Sr doped HA	physical cross-linking	[18]
		agarose	HA	physical cross-linking	[108]
		gelatin	Ce/Zn doped HA	GA	[73]
		gelatin	HA	GA	[44]
				GA	[58]
		gelatin		EDC/NHS	[49]
Nano cellulose	micro-structuring		silica		[98]

### 4.6. Synthetic Polymers

Several synthetic polymers have been investigated for the development of scaffolds for bone or cartilage regeneration, including polyethylene glycol (PEG), polyhydroxyethyl- or polyhydroxypropyl-methacrylate (p-HEMA; p-HPMA), and polyvinyl alcohol (PVA). A summary of them is reported in Table 3.

PEG is a polymer largely used for biomedical application and tissue-engineering [17,102,103,104]. PEG-based scaffolds exhibit good mechanical properties as well as good cyto- and immune-compatibility. Nevertheless, PEG-based cryogels display fast swelling, which can promote homogenous cell spreading within a scaffold [21]. Despite these positive features, PEG can only be proposed in combination with other biopolymers, because PEG’s structure do not allow protein adsorption and cell adhesion, which are basic and fundamental events for tissue reconstruction. As an alternative approach, PEG can be modified with the addition of chemical moieties, which ensure cellular adhesion (i.e., RGD sequences), as reported by Bruns and colleagues [109].

p-HEMA and p-HPMA are two other synthetic polymers which are largely used for medical devices application [110,111] and are also proposed for cartilage tissue repairing [57,112]. They are synthesized by polymerization of the monomers HEMA and HPMA. The corresponding cryogels are generated by cryogenic radical polymerization of the monomers in the presence of N,N’-methylene-bis-acrylamide [57].

p-HEMA cryogels, in particular, present marked tribological properties, as they suffer limited erosion, when they experience strains comparable to those occurring at the joint surface [57]. Cryogels produced with two HEMA derivatives, namely N-vinylformammide (HEMA-NVF) and 1-vinyl-2-pyrrolidinone (HEMA-NVP), showed high water absorption and water retention. Improved water intake leads to a higher incorporation capacity of physiological fluids (i.e., blood or serum). This property relates to higher lubrification of scaffold surfaces with consequent resistance to friction forces, as is the case for articular joints.

Another synthetic polymer adopted for cryogel fabrication is PVA. It is a non-toxic and biocompatible material, suitable for cartilage substitution, as it is able to form scaffolds with mechanical characteristics similar to human cartilage [110,113]. However, like PEG, PVA is also a biologically inert polymer and, therefore, is usually enriched with bioactive and biocompatible components, such as HA [114,115]. The inclusion of HA within PVA cryogels gave stable and homogeneous networks and produced a positive effect on the mechanical behavior of the final construct, as reported for other systems [116].

### 4.7. Inorganic Additives

Cryogel scaffolds proposed for the regeneration of load-bearing tissues are frequently enriched with inorganic additives to improve the properties of the network and better emulate native ECM. In the field of bone regeneration, the addition of these materials offers an opportunity to match the specific mechanical and biological requirements of such complex structures. As mentioned above, silica, graphene, and inorganic salts usually exert a positive stimulus to the complex process of tissue regeneration. Moreover, their use allows for the improvement of the mechanical competence of a scaffold and an increase in the contact surface area [23,71,99].

Several works report the use of calcium phosphate salts; in particular, TCP, biphasic calcium phosphate, and HA are the materials most used in bone rebuilding. The mineral fraction of bone trabeculae is indeed constituted by Ca (II) salts, which, in total, represent about 70% of the bone weight. During the early stage of the mineralization process, Ca (II) ions precipitate on the surface of the ECM proteins, firstly as calcium phosphate, which, after weeks and months of resorption and replacement, becomes amorphous HA crystals. Therefore, the inclusion of HA within a cryogel brings a general improvement in the mechanical resistance and mechanical competence of a scaffold, usually dependent on the apatite content [71,117]. Moreover, as reported for TCP, HA imparts osteoinductive properties on the scaffold and encourages its mineralization. Recently, it has been reported that the use of nano-hydroxyapatite, characterized by nanometric crystallites, may have better osteoconductive properties than micro-sized HA [118]. Specifically, nano-needle or nano-road structures appear to improve these specific properties, due to their similarity to the amorphous native HA, their large contact surface area, and their consequent improved ability to bind proteins [119,120].

**Table 3:** Synthetic polymers employed for bone or cartilaginous tissue reconstruction via cryogel scaffolding.

Principal Cryogel Component	Key Properties	Blended Organic Materials	Inorganic Additives	Cross-Linking Method	Ref.
PEG	hydrophilicity; mechanical resistance			APS/TEMED radical cross-linking of diacrylate PEG	[21]
		Hyaluronic acid; chondroitin sulfate		APS/TEMED radical cross-linking of diacrylate PEG	[20]
		Graphene		graphene oxide	[23]
		γ-polyglutammic acid		APS/TEMED radical cross-linking of PEG-HEMA	[121]
p-HEMA or p-HPMA	hydrophilicity, thermal resistance, tribological properties for articular joint grafts		Zn/Ce substitute hydroxyapatite	APS/TEMED	[113]
				and APS/TEMED radical cross-linking in the presence of MBAAm (N,N′-methylene-bis-acrylamide)	[57]
PVA	cartilage-like mechanical behavior			physical gelation	[122]
				physical gelation	[123]
			HA	physical gelation	[117]
		agarose	Tetraethylorthosilicate; CaCl_2_	physical gelation	[56]
			HA	physical gelation	[115]
			HA	physical gelation	[116]

## 5. Cryogels in the Biological Environment: Physical, Chemical, and Physiological Properties for Scaffold Cellularity and Body Implantability

For effective tissue regeneration, a supporting scaffold requires mandatory characteristics for cellular infiltration as well as for implantation in the physiological environment. To offer a suitable space for cellular colonization, waste–nutrient exchanges, and to guide the differentiation of cells into specialized tissues, specific porosity and stiffness parameters must be respected. Moreover, for surgical implantation into a mammalian organism, it is necessary to obtain a histo-compatible scaffold, which can be naturally integrated into the host environment without any disturbance of the immune system [50,124,125].

In Section 5.1, Section 5.2 and Section 5.3, the influence of the physical and chemical properties of cryogels on scaffold cellularity will be discussed. Moreover, in Section 5.4, the histological integration of cryogel scaffolds in vivo will be analyzed.

### 5.1. Structural Architecture and Mechanical Properties of Cryogels for Cell Hosting

For cellular adaptation, it is fundamental that a scaffold possesses a sponge-like architecture and an adequate porosity to offer an adequate surface for cell anchoring and to allow for the generation of organized tissues.

Another crucial factor is represented by pore interconnectivity, since interconnection along a continuous open maze allows the infiltration and ingrowth of cells, even in deeper and remote portions of a scaffold [15]. Indeed, biomimetic scaffolds, usually employed for bone or articular cartilage reconstruction, display a high level of total open porosity, which is comparable to the porosity range of 30% to 95% found in human bone trabecula or cartilaginous joints [108]. Even the morphology of the porous structure appears to affect cell viability within a scaffold. Generally, a network composed of pores with a uniform size distribution allows deeper cellular infiltration and retention within an entire structure. Nevertheless, a non-uniform pore size distribution appears to increase scaffold cellularity [25], perhaps due to the higher retention which non-homogenous meshes can guarantee. Besides this aspect, pore size also appears to be a relevant parameter to promote cell infiltration and proliferation, even if it is a controversial topic in the literature. It has been reported that adequate cellular infiltration into a cryogel scaffold is reached when the pore size is in the range from 30 μm to 100 μm [15]. Under these conditions, osseous and cartilaginous cells are usually able to proliferate and differentiate [126]. Nevertheless, even if cells are able to colonize scaffolds falling within this pore range, it has been reported that values below 100 μm allow only a partial cellular ingrowth, because they reach a stalemate in the proliferation rate at a certain time of culturing [127]. The critical role of pore size on scaffold cellularity can be related to space limitations, which may hinder cells’ proliferation and, consequently, their differentiation [109]. At the same time, pore size is rationally connected to the ability of a structure to absorb liquids as well as to the diffusion rate through the interconnected channels [50]. These parameters are crucial to allow both homogenous cell seeding and spreading within a scaffold and easy nutrient–waste exchanges with culture medium [41,128]. Therefore, cryogels with adequate porosity may support a more efficient cellular life cycle and may consequently lead to a better differentiation profile [129,130]. However, the pore size topic remains an open issue on which further investigations should be conducted. For instance, the discussion on this topic should include further studies on pores with larger diameters than the above-mentioned ranges, in order to evaluate the effect of larger spans on scaffold cellularity and the ability to retain seeded cells and allow homogenous cell spreading.

Besides scaffold porosity, stiffness also appears to tremendously influence cell anchoring and signaling and, consequently, the differentiation process [16,65,103,129]. The mechanical behavior of a scaffold should replicate the native ECM of the specific cell lineage, in order to exert a differentiation stimulus on seeded cells [129,131]. For example, Liu and colleagues reported that the ECM rigidity can induce osteogenic differentiation of rMSCs (rat mesenchymal stem cells) through the FAK-ERK1/2 signaling pathway, as the focal adhesion kinase (FAK) is an enzyme implied in cellular adhesion, migration, and proliferation [122]. The same dependency of the cellular differentiation response on the stiffness of the scaffold was reported by Bhat and colleagues, who investigated neo-cartilage formation, as well as by Häussling and colleagues, who studied the regeneration of collagenous bone [16,127].

As discussed in previous sections, one of the advantages found in using cryogels for bone and cartilage reconstruction is that the mechanical behavior of a scaffold can be tailored by adjusting and controlling critical fabrication parameters and matrix composition. Tailoring these properties, it is possible to drive signaling pathways and gene expression towards cell adhesion, growing, and differentiation.

### 5.2. Chemical Properties of Scaffolds

Cytocompatibility is of course a mandatory property of cryogel-based scaffolds designed for tissue reconstruction. For this reason, as described in Section 4, natural polymers characterized by cell tolerability and biocompatibility are usually preferred for cryogel fabrication. Moreover, polymers showing a chemical similarity with the native ECM are able to improve scaffold cellularity because it determines the gene expression of proteins typically produced in the matrix of native tissues [89].

A second property of scaffolds that affects cell adhesion and proliferation is matrix hydrophilicity. Indeed, this attribute allows the homogenous infiltration of biological fluids and cells within a scaffold and promotes the adsorption of ECM proteins, such as albumin, fibrinogen, and fibronectin, which are implied in cellular anchoring, proliferation, and differentiation [108,132,133].

The described properties are essential features of cryogels intended for tissue regeneration; however, they are not sufficient for appropriate cell adhesion, which is usually promoted by the presence of specific chemical motifs. Among them, the tripeptide sequence arginine–glycine–aspartate (RGD), largely present in ECM proteins, such as fibronectin and collagen, is one of the most important, as it plays a pivotal role in cell adhesion and proliferation. RGD sequences are recognized by integrins, a kind of binding protein on cellular membranes, which mediate ECM-protein binding in the physiological environment. This bridge between the cellular membrane and the ECM proteins activates biological pathways, which contribute to adhesion, proliferation, and morphological adaptation of cells [62]. In a work published by Koh et al., an increase in chondrogenic phenotype thanks to the addition of RGD sequences in chondroitin-based cryogels was observed. Indeed, the RGD sequences appeared to up-regulate indirectly the expression of early chondrogenic markers in porcine-derivative chondrocytes, such as SOX9, and late chondrogenic markers, such as COL2. Moreover, the expression of type II collagen and consequent collagen accumulation in the scaffold network was observed. These results were related to the high cell viability reached within the scaffold, which was mediated by the adhesion stimulant effect of the RGD sequences [134].

### 5.3. Inorganic Decoration as Adjuvant for Scaffold Cellularity

The cellularity of scaffolds, intended especially for bone reconstruction, is positively influenced by the inclusion, within the polymeric network, of some inorganic components, such as calcium phosphate salts (CP) and silica bio-glasses (SB). As already mentioned, this kind of decoration improves the mechanical competence of a scaffold and acts as a cellular adjuvant. Considering that mesenchymal differentiation is associated with the precipitation of calcium apatites, the inclusion of calcium sources in a scaffold may encourage the secretion of new ECM, thus establishing a faster and more effective healing process at the wound site [118]. Moreover, in a work published by Abueva et al., the adjuvant role of CP crystals for scaffold cellularity was also related to the adsorption of ECM proteins on the CP crystals’ surface [25]. In vitro studies evidenced that the presence of CP had a substantial effect on protein uptake, which influenced the adhesion and proliferation rate of MC3T3-E1 pre-osteoblast cells, which, therefore, resulted in the ability to spread evenly within a scaffold, reaching the inner regions of the network.

**Figure 7:**
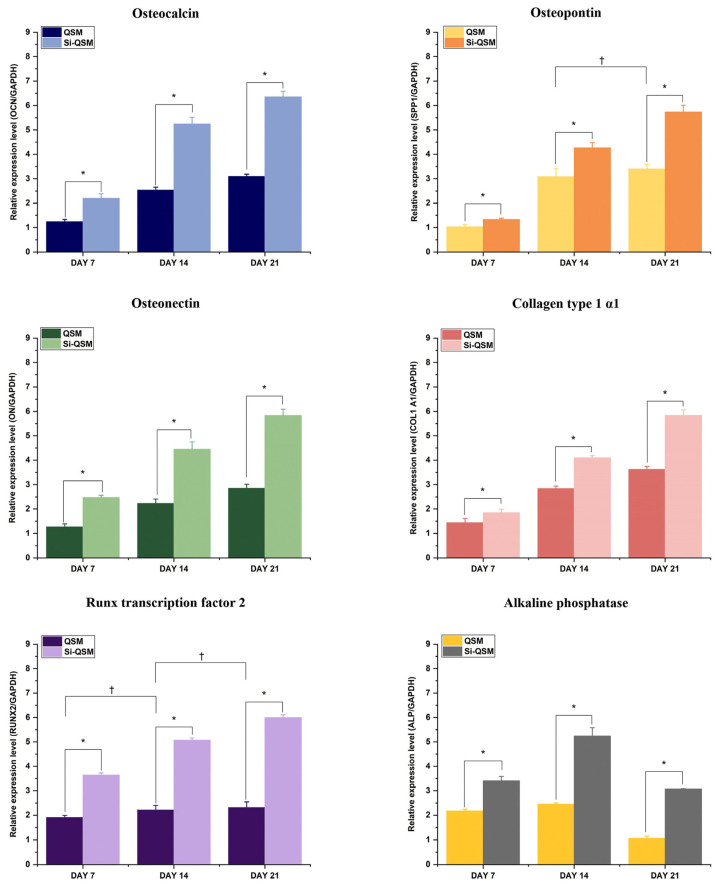
Effect of silica inclusion within quince seed mucilage (QSM) cryogels on the expression of osteocalcin, osteopontin, osteonectin, collagen type I αI (COL1A1), run-related transcription factor 2 (RUNX2), and alkaline phosphatase, evaluated by q-RT PCR. Asterisk indicates *p* < 0.05 and dagger defines the non-significant difference. Reproduced with permission from [28].

Similar behavior was reported for scaffolds enriched with other inorganic materials, such as GO [23] and silica molecules [62], which showed an interesting capacity to induce cell differentiation consequently to the absorption of ECM proteins. Indeed, these scaffolds allowed a remarkable up-regulation of the expression of ECM proteins genes related to osteogenic differentiation, such as osteocalcin, osteopontin, osteonectin, COL1A1, run-related transcription factor 2 (RUNX2), and alkaline phosphatase, as reported in Figure 7 [28]. Interesting osteoinductive properties, associated with antibacterial activity, were also reported for silicon-nitride (SiN)-decorated scaffolds made of gelatin and chitosan [65]. Despite the fact that the biological role of silica in the ossification process remains controversial, SiN inclusion within gelatin–chitosan scaffolds afforded effective bone formation and calcification. These results were related to the strong negative charge of SiN, which causes calcium ion binding and, in this way, promotes apatite deposition. Additionally, the acicular polycrystal microstructure of SiN increased the surface area of the scaffold, thus promoting protein adsorption and, consequently, the bioactivity of the scaffold [65].

### 5.4. Histo-Compatibility and Immune-Tolerance

Tissue regeneration can be achieved following an in vitro or in situ strategy. The second approach consists in the implantation of tissue-specific biomaterials in combination with cells and growth factors at a tissue defect. Such an approach requires the histological integration of the scaffold into the physiological environment. The histo-compatibility of the cryogels is assessed with the implantation of the scaffold in an animal defect model or by subcutaneous implantation [135,136,137].

**Figure 8:**
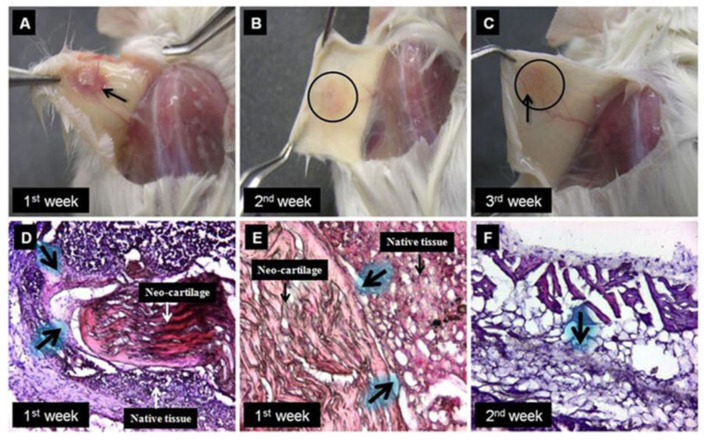
Assimilation of neo-cartilage in the native host tissue. The digital and microscopic images show the sequential assimilation of neo-cartilage with the host native tissue. Implanted neo-cartilage shows some inflammation of the host tissue, as observed after 1st week of implantation (**A**), which disappeared in the 2nd week of implantation (**B**), eventually leading to neo-vascularization (in round circle part shown by arrow) at the site of implantation after 3rd week (**C**). The H&E staining of transverse sections of the site of implantation shows integration of neo-cartilage to the host dermal tissue defined by arrows (**D**,**E**). The neo-cartilage is differentiated from the surrounding tissue (**E**). The vertical section confirms the proper integration of neo-cartilage with the reticular dermis region of dermal tissue (**F**). Reproduced with permission from [16].

Following implantation, the host organism integrates the structure into the native environment, as reported in Figure 8. More specifically, the host responds by producing connective tissue and new blood vessels, which are fundamental for the activation of proliferation and differentiation of mesenchymal cells and, consequently, the formation of new bone or cartilaginous structures [16,106].

In vivo implantation of a scaffold also serves to assess host immune tolerance [92]. Indeed, scaffolds which are suitable for tissue reconstruction must not induce inflammation, degeneration, or necrosis at the site of implantation, so as to not compromise integration at the wound site [76].

## 6. Conclusions

The pioneering field of tissue-engineering recently proposed the use of cryogels to heal critical-sized defects in bone or cartilaginous tissues. Cryogels are innovative polymeric scaffolds, which are formed using a straightforward manufacturing process, which leads to very versatile systems. Thanks to their peculiar porous network, cryogels appear to be able to support cellular growth and differentiation, which, however, can be obtained and aided by opportune selection of scaffold components. Although cryogel-based tissue-engineering appears promising, and much progress has been made in this field, its translation into clinical results is still challenging. A cryogel scaffold must support cellular growing and differentiation, and it should replicate the complex biological and mechanical properties of native bone and cartilage tissues. Proper selection of cryogel components and fine tuning of the fabrication process allows the production of scaffolds with unique and improved physical properties; however, further progress is still needed for a complex bioengineering application, like for the regeneration of functional bone and cartilage tissues. The variability of cryogel compositions, dictated by the precursors used, as well as physical properties such as porosity, pore size distribution, wall thickness, and density provide researchers with a plethora of tools to tune cryogel characteristics in an attempt to match those of native tissues. Moreover, the sponge-like structure of cryogels allows them to change in size rapidly and reversibly, when compressed by external shear stress forces, without permanent deformation. This unique property allows for their potential use as injectable bioscaffolds, without the need for placement through surgery. This feature could have a huge impact on how we design scaffolds for tissue-engineering in the future. Indeed, injectable cryogels could open the way for new and improved therapies with minimally invasive procedures for patients.

Cryogel fabrication can also benefit from the adoption of modern additive manufacturing techniques, such as 3D bioprinting, which may increase the precise personalization of cryogel architecture. These technologies may increase the chances of successful tissue reconstruction, aligning with the principles of modern personalized medicine. Although 3D printing of cryogels is still in its infancy, in our opinion, it shows great potential for the development of complex hierarchical structures able to successfully mimic biological structures.

Once we have reached this goal, the effective clinical use of cryogels needs to be assessed in humans, thus necessitating the move from preclinical animal models to clinical studies. For this reason, rigorous clinical trials and over-extended-time studies are now necessary to assess cryogels’ employability in clinical practice as scaffolds for human implants.

To sum up, cryogel scaffolds show great potential for rebuilding bone and cartilage tissues. Continuous advancements in scaffold design are expected to bring about breakthroughs that are already revolutionizing the field of regenerative medicine. This offers new hope to patients suffering from articular and skeletal non-healing disorders.

## Figures and Tables

**Table 1:** Main advantages and disadvantages of the cross-linking methods most frequently employed for cryogel fabrication.

Cross-Linking Method	Advantages	Disadvantages
Radical cross-linking	Relatively fast cross-linking reaction	Cito-toxicity of unreacted species Employable only with radical-sensitive polymers
GA	Employable on a wide range of bio-polymers containing nucleophiles on their backbone	Cito-toxicity of unreacted species
DVS	Employable on a wide range of bio-polymers containing nucleophiles on their backbone	Cito-toxicity of unreacted species
Carbodiimide coupling	Employable on a wide range of bio-polymers	Cito-toxicity of unreacted species

## Abbreviations

ALP	Alkaline Phosphatase
APS	Ammonium peroxydisulfate
Coll	Collagen
CP	Calcium phosphate
CS	Chondroitin sulfate
Dex	Dextran
DexOx	Oxidized dextran
ECM	Extracellular matrix
FB	Freeze bath
GA	Glutaraldehyde
Gel	Gelatin
GelMA	Methacryloyl gelatin
HAc	Hyaluronic acid
HA	Hydroxyapatite
HEMA	Hydroxyethyl methacrylate
HPMA	Hydroxypropyl methacrylate
OC	Osteocalcin
ON	Osteonectin
OP	Osteopontin
PEG	Polyethylene glycol
RUNX2	Run-related transcription factor 2
SiN	Silicon nitride
TCP	Tricalcium phosphate
TEMED	N,N,N’,N’-tetrametylethylendiamine
UF	Unidirectional freezing
